# Invasive Prenatal Diagnostic Testing for Aneuploidies in Singleton Pregnancies: A Comparative Review of Major Guidelines

**DOI:** 10.3390/medicina58101472

**Published:** 2022-10-17

**Authors:** Eirini Giovannopoulou, Ioannis Tsakiridis, Apostolos Mamopoulos, Ioannis Kalogiannidis, Ioannis Papoulidis, Apostolos Athanasiadis, Themistoklis Dagklis

**Affiliations:** 1 Third Department of Obstetrics and Gynaecology, School of Medicine, Faculty of Health Sciences, Aristotle University of Thessaloniki, 541 24 Thessaloniki, Greece; 2 Access to Genome—ATG, Clinical Laboratory Genetics, 551 34 Thessaloniki, Greece

**Keywords:** invasive prenatal diagnostic testing, chorionic villous sampling (CVS), amniocentesis, indications, technique, complications

## Abstract

Sophisticated screening protocols for genetic abnormalities constitute an important component of current prenatal care, aiming to identify high-risk pregnancies and offer appropriate counseling to parents regarding their options. Definite prenatal diagnosis is only possible by invasive prenatal diagnostic testing (IPDT), mainly including amniocentesis and chorionic villous sampling (CVS). The aim of this comparative review was to summarize and compare the existing recommendations on IPDT from the most influential guidelines. All the reviewed guidelines highlight that IPDT is indicated based on a positive screening test rather than maternal age alone. Other indications arise from medical history and sonography, with significant variations identified between the guidelines. The earlier time for amniocentesis is unequivocally set at ≥15 gestational weeks, whereas for CVS, the earlier limit varies from ≥10 to ≥11 weeks. Certain technical aspects and the overall approach demonstrate significant differences. Periprocedural management regarding Rhesus alloimmunization, virologic status and use of anesthesia or antibiotics are either inconsistent or insufficiently addressed. The synthesis of an evidence-based algorithm for IPDT is of crucial importance to healthcare professionals implicated in prenatal care to avoid unnecessary interventions without compromising optimal prenatal care.

## 1. Introduction 

Prenatal care involves providing a bundle of examinations and guidance to the pregnant woman, to promote education and awareness and prevent or ameliorate adverse outcomes [1]. Detection of genetic abnormalities and birth defects has been a main focus of prenatal screening policies, although screening has also expanded to include potentially preventable adverse outcomes, including preeclampsia, preterm birth and stillbirth [2]. Genetic or birth defects complicate about 3% of births; chromosomal abnormalities include aneuploidies, translocations, deletions and duplications [3]. Major chromosomal abnormalities affect up to 1 in 140 live births [4]. 

Prenatal screening protocols for common aneuploidies, especially trisomy 21, implement various sonographic and/or biochemical markers to produce a risk stratification [2]. During the past few decades, a substantial shift from maternal age alone-based screening to more sophisticated combined screening protocols evolved in clinical practice [5,6]. Different strategies of screening have been proposed in the literature, including integrated, stepwise sequential or contingency screening that are available in the first and/or second trimesters of pregnancy [7,8]. Aneuploidy screening has radically changed following the introduction of cell-free DNA (cfDNA) testing, which has been validated as a highly accurate screening tool, especially in singleton pregnancies [9]. 

According to its definition, screening provides only a risk estimate and does not constitute a diagnosis. Definite diagnosis is only achieved by cytogenetic analysis of cells obtained through invasive prenatal diagnostic testing (IPDT); the latter includes chorionic villous sampling (CVS) and amniocentesis as well as fetal blood sampling (FBS) under specific indications [10,11]. The first diagnostic amniocentesis for trisomy 21 dates back to 1968 [12], and the description of the technique preceded this by several years [13,14]. Amniocentesis became the gold standard of prenatal diagnosis during the 1970s, and CVS was introduced a decade later [15,16]. Since then, as experience in IPDT has accumulated, several alterations were implemented on indications, timing, technical aspects and cytogenetic analysis techniques; procedure-related complications were also described [11]. The choice of the procedure is affected by both the operator’s expertise and the individual patient’s preferences that are reflected in decision making. IPDT should be undertaken by appropriately trained healthcare professionals, taking into consideration the inter-operator variability, as well as the associated cost [17,18,19].

Several medical societies have developed guidelines to address the issues related to IPDT and guide clinical practice, which has substantially evolved during the past few decades. As there are considerable differences in recommended practices and approaches, usually affected by cost-effectiveness analyses and associated healthcare policies, we decided to undertake this review to summarize and compare the recommendations provided by influential medical societies with regard to IPDT for fetal genetic defects and underline potential agreements and disagreements. 

## 2. Evidence Acquisition 

The most recently published guidelines from seven medical societies on IPDT were retrieved, and a descriptive review was performed. We included recommendations from the National Society of Genetic Counselors (NSGC 2013) [7], the American College of Obstetricians and Gynecologists and the Society for Maternal-Fetal Medicine (ACOG-SMFM 2016) [20], the International Society of Ultrasound in Obstetrics and Gynecology (ISUOG 2016) [21], the Human Genetics Society of Australasia and the Royal Australian and New Zealand College of Obstetricians and Gynaecologists (HGSA—RANZCOG 2018) [22], as well as the Royal College of Obstetricians and Gynaecologists (RCOG 2021) [23].

An overview of recommendations is presented in Table 1 (indications for IPDT), Table 2 (amniocentesis), Table 3 (chorionic villus sampling) and Table 4 (periprocedural management). Of note, the RCOG guideline does not make any reference to the indications of IPDT.

## 3. Indications for Invasive Prenatal Diagnostic Testing 

It is well-documented that prenatal screening for fetal aneuploidies should be offered to all women [24]. Screening options are delineated by guidelines and local standards. However, as already mentioned, the result of a screening test does not constitute a definite diagnosis. Definite diagnosis requires IPDT, which is generally reserved for high-risk women. 

### 3.1. High-Risk Groups for Fetal Aneuploidy 

Several reviewed guidelines recommend offering IPDT when a screening test is positive or above predetermined cut-off values, including variable protocols for combined screening (NSGC, ACOG-SMFM, ISUOG, HGSA-RANZOG). Moreover, abnormal ultrasound findings represent another common indication for IPDT (NSGC, ACOG-SMFM, ISUOG); however, specific ultrasound findings that require diagnostic testing differ among these guidelines. In particular, ACOG-SMFM does not specify the ultrasound findings that should prompt investigation with IPDT. However, it is stated that the risk fluctuates based on the number and type of the anomalies. Wladimiroff et al. described the findings from karyotyping a total of 170 fetuses with specific structural defects or fetal growth restriction, polyhydramnios and fetal hydrops [25]. The majority of the cases included either one major defect (including cardiac defects, duodenal atresia, omphalocele or cystic hygroma), multiple minor defects or rare deficits; a chromosomal abnormality was detected in 20.5% of the analyzed cases [25]. ISUOG defines abnormal ultrasound findings as the recognition of a structural anomaly indicative of chromosomal abnormalities, but does not provide specific information on these abnormalities. Additionally, the NSGC states that a nuchal translucency (NT) measuring ≥ 3 mm or above the 95th percentile should be followed by IPDT. Souka et al., in a review on the association between increased NT and major fetal abnormalities in chromosomally normal fetuses, found that the prevalence of abnormalities increases with NT thickness (1.6% in NT < 95th percentile, 2.5% for NT between 95th and 99th percentiles, up to 45% for NT ≥ 6.5 mm) [26]. 

### 3.2. Past History 

A personal history of a previous child or fetus diagnosed with chromosomal abnormality poses an independent indication for invasive testing (ACOG-SMFM, ISUOG). This is derived and supported by data reporting increased risk of recurrence in subsequent pregnancies [27]. Warburton et al. investigated the risk for trisomy recurrence combining the data from two large databases; the risk for trisomy 21 recurrence is higher than the expected based on maternal age, when the first occurrence was after 30 years of age [27]. Nevertheless, the risk of recurrence of a viable trisomy is multiplied by 1.6 to 1.8 after a previous history of trisomy 21, 18 or 13, independently of the viability of the fetus [27]. 

As far as parental status is concerned, known carrier status for a balanced chromosomal translocation or inversion or parental aneuploidy or mosaicism for aneuploidy justify further diagnostic testing according to ACOG-SMFM and ISUOG. Parental carrier status that has been diagnosed after a history of an affected child dramatically increases the risk for chromosomal abnormality in the current pregnancy, compared to cases incidentally diagnosed, with no previous history [28]. More specifically, the relevant risks are 5–30% compared to 0–5% for translocations and 5–10% compared to 1–3% for inversions [28]. 

### 3.3. Maternal Request 

NSGC, ACOG-SMFM and HGSA-RANZCOG support offering the option of IPDT to all women, irrespective of age or other risk factors. According to ISUOG, however, IPDT is not justified based solely on maternal request and should be offered only after extensive counseling by an expert. The reasoning behind offering to all women the option of invasive testing is supported by the fact that using array comparative genomic hybridization (array-CGH) technology in pregnant women, irrespective of age, with normal ultrasound and karyotype, the risk of finding a pathogenic copy number variant is >1% [29,30]. Taking into consideration the distribution of maternal age, which gradually increases, the reduced complication rates of invasive procedures and the personal beliefs and expectations of each woman for their pregnancy, an individualized approach is encouraged to allow for informed decisions regarding invasive testing [31,32]. 

### 3.4. Maternal Age 

ACOG-SMFM, ISUOG and HGSA-RANZCOG agree that maternal age as a standalone criterion is not an indication for invasive prenatal testing. The concept of maternal age-based strategies for IPDT has been re-evaluated in the past two decades [31]. There is a shift towards screening-based risk stratification and maternal age is co-evaluated among other factors, derived from screening protocols [32,33,34,35]. This shift is justified, as screening strategies are evolving to become more sensitive and intend to minimize procedure-related risks and costs, associated with invasive testing [31]. 

### 3.5. Assisted Reproduction Techniques 

ISUOG recommends against routine IPDT following IVF or ICSI, in the absence of other risk factors; however, in the context of ICSI due to oligospermia, it recommends counseling of the couple for the higher risk of chromosomal abnormalities associated with infertility in the male offspring. Bonduelle et al. investigated 1586 fetuses conceived by ICSI and found a significantly higher rate of inherited chromosomal abnormalities in these cases, compared to the general population (1.4% vs. 0.3%) [36]. The majority were attributed to the male partner and associated with sperm quality, initially necessitating ICSI [36]. The other guidelines do not make any relevant recommendation.

### 3.6. Cell-Free DNA

cfDNA is based on the analysis of circulating fractions of fetal DNA in maternal serum to achieve prenatal screening for aneuploidies [37,38]. cfDNA is a screening and not diagnostic test and therefore should not substitute IPDT (NSGC). The role of cfDNA is either as a first-tier screening tool for fetal aneuploidy or as second-tier screening after a positive screening result (derived, for example, from combined first trimester screening) and before IPDT is undertaken (HGSA-RANZCOG). The latter approach may place some originally high-risk women at a low-risk level, and as they may avoid invasive testing, some cases with aneuploidy may be missed [39]. In any case, a positive result of a non-invasive prenatal test (NIPT) should be referred for IPDT (NSGC, ACOG-SMFM, ISUOG, HGSA-RANZCOG). Moreover, low fractions of cfDNA in maternal serum (less than 4%) may lead to an inconclusive test result, referred to as a “no call” result [24]. HGSA-RANZCOG suggests IPDT among other options (detailed ultrasound follow-up or combined screening if not already performed or even repeat cfDNA, on the grounds of a higher risk of aneuploidy). Pergament et al. analyzed cfDNA in 1051 pregnancies and found that in cases of aneuploidy, a percentage as high as 16% did not return a result; in half of these cases, the fetal fraction was below the 1.5th percentile compared to normal euploid samples [40]. Moreover, a “no-call” result due to a low fraction of cfDNA was associated with significantly higher odds for aneuploidy (OR: 5.7; 95% CI: 2.5–13.1), compared to samples within the normal range [40]. Several studies have demonstrated that fetal fraction is significantly higher in trisomy 21 and lower in trisomies 13, 18 and triploidies, compared to euploid pregnancies [41,42]. 

Of note, there may be additional contributing factors to a “no-call” result in the context of aneuploidy, other than low fetal fraction, that have not been extensively investigated or understood [43]. Particularly, variables such as maternal body mass index, maternal age, gestational age, medications, ethnicity and conception through assisted reproduction techniques may also interfere with the fraction of cfDNA, acting as significant confounders to the interpretation of the results [42,44,45]. Repeating the cfDNA test at a later gestational age in these cases is reasonable (as cfDNA fraction increases with advancing gestational age), but may delay definite diagnosis [43,45,46]. Previous screening results and ultrasound findings should be taken into account in the interpretation of noninformative results and clinical decision making [42].

Another concern is that confined placental mosaicism (CPM) is a common cause of a false positive cfDNA result associated with a normal euploid fetus [47]. On the other hand, CVS is associated with an incidence of about 2% of cell mosaicism, and only 13% of those cases correspond to true fetal mosaicism, as confirmed by amniocentesis [48]. Therefore, since CPM may per se be the reason for an abnormal cDNA result, there are thoughts about the potential superiority of amniocentesis over CVS to provide a definite diagnosis in such cases [49]; however, this issue is not addressed by the reviewed guidelines. 

## 4. Available Techniques for Invasive Prenatal Diagnostic Testing 

### 4.1. Amniocentesis 

#### 4.1.1. Timing 

All guidelines agree that amniocentesis should be performed only after 15 completed gestational weeks. The reason for this recommendation is the well-documented higher risk of procedure-related complications in cases of early amniocentesis, including pregnancy loss, fetal congenital defects and membrane rupture at an earlier gestational age [50,51,52,53].

#### 4.1.2. Technical Aspects 

Amniocentesis involves the insertion of a needle system through the abdominal wall into the amniotic cavity to obtain amniotic fluid for genetic analysis [54]. The sample contains fetal exfoliated cells, transudate, fetal urine and secretions [54]. The maximum caliber of the needle for amniocentesis is a key technical aspect. ACOG-SMFM recommends a 22-gauge needle, whereas according to the ISUOG guidelines, either a 20 G or 22 G needle may be used; a larger caliber needle is associated with quicker fluid aspiration without increasing the risk of intrauterine bleeding [55]. Based on the findings of previous studies, transplacental needle passage increases the risk of contamination with blood [56,57,58]. Therefore, the passage of the needle through the placenta or at the placental cord insertion site should be avoided, unless it is the only alternative to safely access an adequate amniotic fluid pool. ACOG-SMFM and ISUOG further underline the importance of such an approach, especially for Rhesus-negative women, due to the potentially higher incidence of feto-maternal hemorrhage and alloimmunization [58]. ISUOG, ACOG-SMFM and RCOG highlight the necessity of continuous ultrasound visualization during the procedure. Of note, the other guidelines do not make any relevant recommendation.

#### 4.1.3. Maternal Cell Contamination 

ACOG-SMFM, ISUOG and RCOG are the only guidelines that comment on the possibility of maternal cell contamination in the sample retrieved from amniocentesis. The frequency of this condition greatly varies among different series and is higher in cases of transplacental passage, need for second needle insertion, operator’s lack of experience and blood staining of the amniotic fluid [59,60,61]. In order to avoid maternal contamination, ACOG-SMFM and ISUOG encourage the disposal of the first 1–2 mL that are aspirated. RCOG states that the possibility of maternal contamination during amniocentesis is 1–2% but does not provide any further guidance on this matter. 

#### 4.1.4. Complications

Like any interventional procedure, amniocentesis is not without complications. In fact, this is a key issue in counseling, as parents need to decide based on the trade-off between the advantage of diagnosis and the associated risks. Fetal loss, postprocedural fluid leakage, fetal defects and chorioamnionitis are the main concerns following amniocentesis; the rate of estimated procedure-related complications varies among studies and, therefore, between different guidelines. According to ACOG-SMFM, amniocentesis has an overall complication rate of 0.1–0.3%, as suggested by recent studies [62,63,64]. Akolekar et al. conducted a systematic review and metanalysis on procedure-related fetal loss, including 42,716 women undergoing amniocentesis [62]. The procedure-related loss was estimated at 0.11%. Similarly, Odibo et al. provided data from a single-center retrospective analysis of 11,746 women subjected to amniocentesis, and the associated risk was estimated at 0.13% [63]. Moreover, Caughey et al. reported a fetal loss rate of 0.27% following amniocentesis [64]. 

NSGC agrees that fetal loss ranges between 0.1% and 0.3%, complying with the lower risk estimates [65]. On the other hand, ISUOG and RCOG refer to slightly higher miscarriage rates of 0.1–1% and <0.5%, respectively. A recent meta-analysis on procedure-related losses, including 64,901 amniocentesis and 19,000 CVS, updated the procedure-related fetal losses at 0.35% for both procedures [66]. According to ISUOG, fetal loss increases with multiple needle insertions, blood contamination of the amniotic fluid and the presence of an underlying fetal abnormality [67,68]. 

Rupture of membranes is another potential complication of amniocentesis, encountered in 1–2% of cases [53,69,70,71]. Congenital limb malformation is another concern; NSGC estimates the possibility of clubfoot to be less than 1% [53,69,72]. Post-procedural infection of the fetal membranes, clinically presenting as chorioamnionitis, is quite rare, with an incidence of <0.1%, as described by ISUOG. Other severe feto-maternal complications have been only occasionally reported and considered rare, such as sepsis, maternal visceral injury or fetal injury. Of note, experience, reflected on the number of procedures performed annually by each operator, also plays a critical role in the incidence of procedure-related complications [73,74,75]. Baker et al. retrospectively investigated the effect of multiple variables on procedure-related fetal losses after CVS and amniocentesis and found a positive association between increasing experience and lower procedure-related risks, highlighting that actual risks may be lower than those initially estimated and on which routine counseling is based [76]. Therefore, counseling should be accordingly reformed to incorporate the updated available evidence on associated risks. 

### 4.2. Chorionic Villous Sampling 

#### 4.2.1. Timing

CVS is the only available diagnostic test during the first trimester (NSGC, ACOG-SMFM, ISUOG, HGSA-RANZCOG, RCOG), as early amniocentesis before 14 weeks is unanimously discouraged due to an unacceptably higher rate of complications [50,51,52,53]. However, the optimal gestational age after which CVS should be performed is not clear; NSGC, ACOG-SMFM, ISUOG and RCOG set the safe limit to perform CVS at 10 weeks, while HGSA-RANZOG recommends against CVS before 11 completed gestational weeks due to a higher risk of limb defects. RCOG also states that CVS should ideally be performed after 11 completed weeks, as the suboptimal development of the trophoblastic tissue increases the technical difficulty of the procedure at an earlier gestational age.

#### 4.2.2. Technical Aspects 

CVS includes the introduction of a needle system through the abdomen (transabdominal) or the cervix (transcervical) to retrieve chorionic tissue from the developing placenta for genetic analysis [19]. CVS can be safely carried out by either a transabdominal or transcervical approach by continuous US guidance (ISUOG, ACOG-SMFM, RCOG). ISUOG makes a recommendation on the needle size, recommending either a single needle of 17–20 G or a two-needle system with outer needle size of 17/19 G and inner needle size of 19/20 G. The variation in the technique and the needle size in clinical practice is highlighted by the study of Carlin et al. that reviewed the individual preferences in U.K. practice [77]. Like the amniocentesis technique, apart from ISUOG and RCOG, the other guidelines do not make any relevant recommendation.

#### 4.2.3. Complications

Regarding the risk of pregnancy loss, CVS is comparable to mid-trimester amniocentesis, when performed by experienced operators. ACOG-SMFM reports a procedure-related loss of 0.22%, while ISUOG provides an estimate for fetal loss that greatly varies between 0.2% and 2% [62], underlining the absence of well-designed randomized controlled studies. In the systematic review and meta-analysis conducted by Akolekar et al., which included 8899 CVS procedures, the risk was estimated at 0.22% [62]. NSGC sets the risk between 0.5% and 1% and RCOG below 0.5%. Odibo et al. retrospectively evaluated the risk of pregnancy loss in 5148 CVS procedures and found a risk estimate of 0.2% and 0.5% for transabdominal and transcervical procedures, respectively [78]. Interestingly, the risk was not statistically different from the background risk in the control group [78]. CVS may also be associated with vaginal bleeding in 10% or even 30% of cases after a transcervical approach [79,80]. In addition, amniotic fluid leakage and intra-amniotic infection are encountered much less often (<0.5% and 1–2 per 3000 cases, respectively), according to ACOG-SMFM and ISUOG [79]. As far as maternal safety is concerned, no cases of severe maternal adverse outcomes have been described following CVS, or at worse, they are very rare [23].

#### 4.2.4. Fetal Blood Sampling 

FBS entails access to fetal circulation in order to obtain a blood sample for analysis. Fetal blood is obtained via puncture of the umbilical vein, and therefore, also referred to as “cordocentesis”. The umbilical vein can be accessed either at the cord (cord insertion or independent loop) or even at its intrahepatic portion, according to placental location [81]. FBS is the first-line option for the hematologic assessment of the fetus in cases of severe anemia or thrombocytopenia. However, its role in prenatal genetic diagnosis is limited [81]. The only guideline that refers to FBS as a means of prenatal diagnosis is ISUOG. FBS is indicated in cases of mosaicism of the sample obtained from invasive testing, to exclude true fetal mosaicism. Concerning indications, the ISUOG guideline refers to investigation of mosaicism solely after amniocentesis with no referral to CVS. According to ISUOG, cordocentesis for FBS can be performed from 18 completed weeks [81], and the risk of procedure-related pregnancy loss is 1–2% [82]. However, this risk increases with gestational age under 24 weeks, possibly due to associated structural malformations or fetal growth restriction [83,84]. According to ISUOG, the optimal technique includes the use of a 20–22 G needle that is inserted to the cord, through the abdomen, under simultaneous ultrasound visualization. 

## 5. Periprocedural Management 

The optimal management of a woman who has an indication for invasive diagnosis is minutely delineated by the ISUOG and RCOG guidelines (ACOG-SMFM makes recommendations only for transmittable diseases).

- Rhesus status

According to ISUOG, Rhesus status of the mother, along with the existence of alloantibodies in the maternal serum, is a prerequisite before the procedure, in order to administer immunoglobulin in Rhesus-negative women. Anti-D administration, when indicated, should not delay more than 72 h from the procedure (ISUOG). Additionally, according to ISUOG, immunization could be omitted, if the Rhesus status of the presumed father has been confirmed as negative. RCOG states that Rhesus-negative women should be provided appropriate aftercare for immunization but does not offer any further guidance.

b. Transmittable diseases

There is controversy regarding the need for screening for maternal blood-borne diseases before IPDT. In particular, ISUOG recommends against routine screening, based on available data indicating that vertical transmission is an unlikely event and may only affect pregnancies with high maternal viral load [85,86]. ISUOG also states that when invasive diagnosis is indicated, placental penetration should be avoided in women known to be HIV-, HBV- or HCV-positive [85]. On the other hand, RCOG emphasizes the need for universal screening or counseling for women who are unwilling to undergo virology testing, for the potential of vertical transmission during the procedure. Data mainly pertain to amniocentesis, as studies for vertical transmission of chronic infection in CVS are lacking. 

### 5.1. HIV

ACOG-SMFM states that HIV transmission is not increased in women receiving antiretroviral therapy and whose viral load is undetectable. The ISUOG recommendation agrees that HIV-positive women on highly active antiretroviral therapy (HAART) are not at increased risk for vertical transmission [87], even if the viral load is high, as far as HAART is initiated at least two weeks before the procedure [88,89]. Postponement of the procedure until the viral load is undetectable is also suggested by ACOG-SMFM [85,90]. According to RCOG, IPDT should be withheld until HIV results are available. For HIV-positive women under HAART, therapy optimization to aim for undetectable viral load is reasonable before any intervention, to minimize the risk of vertical transmission [91].

### 5.2. HBV

ACOG-SMFM aligns with the low incidence of neonatal infection in HBV-infected mothers with low viral load but also underlines the relevant gap in the literature for exposed cases [92]. RCOG considers the risk for vertical transmission for HBV infection to be low unless the viral load exceeds the threshold of ≥7 log_10_ copies/mL; individualized assessment of risk is thus recommended. 

### 5.3. HCV

ACOG-SMFM comments on the paucity of knowledge regarding vertical transmission of HCV but states that the risk is presumably low [93]. ISUOG also underlines the importance of counseling about the limited data on fetal infection for CVS or FBS, compared to amniocentesis [85], while recommending that the option of non-invasive testing should be considered in women with transmittable diseases, in the absence of adequate high-quality evidence [69]. RCOG comments on the absence of evidence of HCV vertical transmission [93].

### 5.4. SARS-CoV-2 

During the SARS-CoV-2 pandemic, another issue is dealing with pregnant women who tested positive and are in need of IPDT. Chronologically, the majority of the included guidelines (except RCOG) preceded the pandemic and hence, relevant recommendations are not available. The best available data, although limited, suggest that invasive testing is safe in COVID-19-positive pregnant women and the risk of vertical transmission is considered to be low [94].

c. Aseptic technique

To minimize the risk of infection, ACOG-SMFM, ISUOG and RCOG highlight the importance of a sterile technique. Of note, ISUOG and RCOG encourage the use of a sterile containment bag for the ultrasound probe and a separate sterile gel; decontamination of the ultrasound probe after each procedure is an alternative to the sterile bag (ISUOG). Moreover, both ISUOG and RCOG underline the significance of skin sterilization. ISUOG suggests skin decontamination with a chlorhexidine or iodine disinfectant solution and the use of sterile drape, and for transcervical CVS, the use of a sterile speculum and antisepsis of the cervical and vaginal mucosa.

d. Local anesthesia

Application of a local anesthetic is not recommended in amniocentesis [95,96] and is considered optional in transabdominal CVS by ISUOG [95,96,97]. Data for CVS through transcervical approach are not available. For FBS, the ISUOG recommendation follows that for transabdominal CVS [81]. The other guidelines do not make any relevant recommendation.

e. Other considerations

ISUOG explicitly states that ultrasound should be performed routinely both prior and after completion of invasive diagnosis. Pre-procedural ultrasound aims at confirming viability and gestational age and assessing the location of the placenta and the amount of amniotic fluid [97]. Post-procedural ultrasound should include fetal heart rate, assessment of the placenta and amniotic fluid to exclude complications associated with placental hematoma or post-procedural fluid leakage and may take place immediately after the procedure or even days later, based on routine practice [67]. 

Routine antibiotic prophylaxis or any other medical therapy are generally not recommended. However, RCOG states that if there are clinical signs of chorioamnionitis or a macroscopic appearance of the amniotic fluid consistent with microbial infection, analysis of the sample and initiation of antibiotic therapy is recommended. 

Based on the results of studies investigating other invasive percutaneous procedures [98], women receiving thromboprophylaxis or prophylactic low-dose aspirin are not advised to discontinue the regimen, according to ISUOG.

f. Counseling

All guidelines underline the significance of proper genetic counseling in patients at high risk of aneuploidy provided by an appropriately trained healthcare professional. Pretest counseling should precede an invasive procedure to address the risks, the benefits, the technical aspects and the available options in a nondirective manner. In cases of mosaicism, genetic counseling is recommended to discuss the possibility of fetal involvement and allow for offering options (ACOG-SMFM, ISUOG). Counseling should be based on evidence regarding the possibility of true fetal mosaicism. Malvestiti et al. investigated the incidence of mosaicism in 60,437 CVS samples and found a percentage of 2%; of those, 1001 cases were subjected to amniocentesis [48]. 

## 6. Conclusions

Overall, the included guidelines all support the availability of definite diagnostic testing to every pregnant woman after appropriate counseling and recommend IPDT based on a positive screening result. Maternal age alone should not constitute an indication for IPDT. There is, however, controversy among these guidelines on the additional indications that may prompt diagnostic testing, such as patient history (personal or familial), known carrier status of either parent, conception via ART or specific ultrasound findings, leading to substantial differences in clinical practice. 

There is general agreement on the appropriate timing for amniocentesis, which is set at 15 weeks, whereas CVS is mostly recommended from 10 weeks, with the exception of HGSA-RANZOG, which recommends that it is performed after 11 weeks. The recommendations regarding counseling on complications rates are based on different studies and thus there are certain differences. However, there is a clear trend to counsel patients that complications are nowadays rarer than initially reported, and a significant decrease with advancing operators’ experience is highlighted. Data on periprocedural management such as Rhesus alloimmunization, virologic status, the role of anesthesia and antibiotic administration are either inconsistent or insufficiently addressed. 

The major strength of this comparative review is the synthesis of the most influential guidelines on IPDT. However, there are certain limitations. First, we opted not to search for all available guidelines systematically, because we intended to compare recommendations only from major medical societies. Thus, we included five guidelines, published in the English language, in our comparisons. Finally, the publication dates of the guidelines differ; some discrepancies may be due to the fact that some of the guidelines were developed up to nine years before and therefore may be partially outdated. 

Amniocentesis and CVS are common procedures. However, our study has demonstrated that current national guidelines are in many aspects contradictory and incomplete, while international guidelines may not be able to be fully implemented in all settings due to different cultural and economic conditions. Thus, the development of a standardized, evidenced-based model for the efficient and safe use of IPDT is of paramount importance. Such an approach should help reduce the heterogeneity in local practices and offer a high level of prenatal care to all women, irrespective of national boundaries. The present review aimed to identify similarities and dissimilarities on IPDT and also highlight potential fields for future research. As knowledge accumulates, it becomes evident that the enormous amount of information should be properly guided and communicated to healthcare professionals in prenatal care with the aim to promote the health and well-being of every mother and her fetus. 

## Figures and Tables

**Table 1:** Summary of recommendations on the indications for invasive prenatal diagnostic testing.

	NSGC	ACOG-SMFM	ISUOG	HGSA-RANZCOG	RCOG
Issued	2013	2016	2016	2018	2021
Title	NSGC Practice Guideline: Prenatal Screening and Diagnostic Testing Options for Chromosome Aneuploidy.	Prenatal Diagnostic Testing for Genetic Disorders.	ISUOG Practice Guidelines: Invasive procedures for prenatal diagnosis.	Prenatal screening and diagnostic testing for fetal chromosomal and genetic conditions.	Amniocentesis and chorionic villus sampling.
Pages	12	14	13	35	15
References	44	74	106	47	55
Indications for IPDT	Positive screening result. Ultrasound findings (NT > 3.0 mm or >95th percentile).	Positive screening result. Ultrasound findings (not specified).	Positive screening result. Ultrasound findings (structural defects associated with chromosomal abnormalities).	Positive screening result.	Not discussed
Past History	Not discussed	Previous child or fetus with chromosomal aneuploidy. Known parental carrier status.	Previous child or fetus with chromosomal aneuploidy. Known parental carrier status.	Not discussed	Not discussed
Maternal request	Available to all women.	Available to all women.	Only under specific circumstances.	Available to all women.	Not discussed
Maternal age	Not discussed	Advanced maternal age does not justify invasive testing.	Advanced maternal age does not justify invasive testing.	Available to all women irrespective of age.	Not discussed
Assisted Reproduction Techniques (ART)	Not discussed	Not discussed	IVF or ICSI is not an indication for IPDT.	Not discussed	Not discussed
Cell-free DNA	A positive cfDNA result.	A positive cfDNA result.	A positive cfDNA result.	A positive or a “no call” cfDNA result.	Not discussed

ICSI: intracytoplasmic sperm injection; IVF: in vitro fertilization; IPDT: invasive prenatal genetic diagnosis; NT: nuchal translucency.

**Table 2:** Summary of recommendations on amniocentesis.

	NSGC	ACOG-SMFM	ISUOG	HGSA-RANZCOG	RCOG
Issued	2013	2016	2016	2018	2021
Timing	≥15 weeks	≥15 weeks	≥15 weeks	≥15 weeks Not recommended before 14 weeks.	≥15 weeks
Technique	Not discussed	Continuous ultrasound guidance. Sterile technique. Needle size 22 G.	Continuous ultrasound guidance. Aseptic technique. Maximum needle size 20–22 G. Avoid placenta and placental cord insertion site, especially in Rhesus-negative women.	Not discussed	Continuous ultrasound guidance. Aseptic technique.
Complications	Fetal loss, limb defects, membrane rupture.	Fetal loss, vaginal spotting, membrane rupture.	Fetal loss, chorioamnionitis, membrane rupture. Fetal injury, maternal complications (rare).	Not discussed	Fetal loss, severe infection, fetal injury, maternal visceral injury.
Maternal cell contamination	Not discussed	Discard the first 1–2 mL.	Discard the first 2 mL.	Not discussed	Risk 1–2%.

**Table 3:** Summary of recommendations on chorionic villus sampling.

	NSGC	ACOG-SMFM	ISUOG	HGSA-RANZCOG	RCOG
Issued	2013	2016	2016	2018	2021
Timing	Not discussed	≥10 weeks	≥10 weeks	≥11 weeks	≥10 weeks If possible after 11 completed weeks, when technically easier.
Technique	Not discussed	Transabdominal or transcervical approach. Continuous ultrasound guidance.	Transabdominal or transcervical approach. Continuous ultrasound guidance. Aseptic technique.	Not discussed	Continuous ultrasound guidance. Aseptic technique.
Complications	Fetal loss, limb defects (especially before 10 weeks). Procedure-related complications comparable with amniocentesis, only in experienced centers.	Fetal loss, vaginal bleeding, limb defects (especially before 10 weeks).	Fetal loss, vaginal bleeding, amniotic fluid leakage, chorioamnionitis.	Not discussed	Fetal loss, severe infection, fetal injury, maternal visceral injury.

**Table 4:** Summary of recommendations on periprocedural management at invasive prenatal diagnostic testing.

	NSGC	ACOG-SMFM	ISUOG	HGSA-RANZCOG	RCOG
Issued	2013	2016	2016	2018	2021
Periprocedural management	Not discussed	Not discussed	Provide a detailed report. Ultrasound check for fetal heart rate, hematoma and amniotic fluid after the procedure.	Not discussed	Need to have a written consent form before invasive testing.
Rhesus alloimmunization	Not discussed	Not discussed	Check Rhesus and alloantibodies. Administer anti-D immunoglobulin in Rhesus-negative women within 72 h, unless there is proof that the alleged father is Rhesus-negative.	Not discussed	Inform patients of aftercare, including Rhesus immunization in non-sensitized Rhesus-negative women.
Blood-borne viral diseases	Not discussed	Routine screening not recommended. Recommendations apply to known chronic infections.	Routine screening for transmittable viral diseases not recommended.	Not discussed	Universal screening for blood-borne viral disease is recommended and is performed by review of previous records.
HIV, HBV, HCV	Not discussed	Low incidence of HBV vertical transmission with low viral load. Risk for HCV vertical transmission presumably low. Low risk of HIV vertical transmission in women under antiretroviral therapy and undetectable viral load. Optimally postpone invasive testing until viral load is below detection cut-off. Data for CVS are limited.	Noninvasive testing is preferable to invasive procedures in known HCV-, HBV- or HIV-positive status. If invasive testing is performed, avoid the placenta. In cases of HBsAg (+)/HbeAg (−) or HIV (+) in HAART, the risk of vertical transmission is not increased.	Not discussed	HBV or HCV infection is not a contraindication for invasive testing. Minimal risk for vertical transmission for HBV and low viral load and no proven risk for HCV. Withhold invasive testing until HIV results available.In cases of HIV (+) under HAART, the risk is low. Optimally, postpone invasive testing until viral load is below detection cut-off.
Aseptic technique	Not discussed	Sterile technique. Details not specified.	Sterile gloves, gauzes, needles, forceps. Skin decontamination (chlorhexidine or iodine). Sterile drape. Sterile bag for ultrasound probe or probe disinfection. Sterile gel. Sterile speculum and disinfection of cervical and vaginal mucosa for transcervical CVS.	Not discussed	Skin decontamination. Sterile bag for ultrasound probe. Sterile gel.
Anesthesia	Not discussed	Not discussed	Local anesthesia not recommended for amniocentesis. There are no available data for transcervical CVS. Consider local anesthesia for transabdominal CVS to reduce discomfort and maternal movement.	Not discussed	Not discussed
Ultrasound evaluation	Not discussed	Not discussed	Pre-procedural (HR, GA, placenta, amniotic fluid). Post-procedural (HR. placenta, amniotic fluid). Check for complications immediately after or even days after.	Not discussed	Not discussed
Antibiotics	Not discussed	Not discussed	Antibiotic prophylaxis not recommended.	Not discussed	Consider antibiotic therapy in cases of purulent or cloudy amniotic aspirate or in the presence of clinical chorioamnionitis.
Thromboprophylaxis	Not discussed	Not discussed	Discontinuation of the regimen in women receiving thromboprophylaxis or low dose aspirin is not recommended.	Not discussed	Not discussed
Counseling	Refer for genetic counseling when there are concerns in decision making.	Offer pretest counseling. Nondirective counseling. Refer to genetic counseling after a suspected diagnosis of aneuploidy or in complex cases of mosaicism.	Offer pretest counseling. Genetic counseling in cases of sample mosaicism.	Offer individualized counseling. Genetic counseling in high-risk women	Pretest counseling by appropriately trained professionals.

HIV: human immunodeficiency virus; HBV: hepatitis-B virus; HCV: hepatitis-C virus; CVS: chorionic villus sampling; HAART: highly active antiretroviral therapy; HR: heart rate; GA: gestational age.

## Data Availability

All the data used for this article are publicly available.
